# Supplementary material for: Patterns of Intron Gain and Loss in Fungi
Source: PLoS Biol. 2004 Nov 30;2(12):e422. doi: 10.1371/journal.pbio.0020422 (PMC532390; doi:10.1371/journal.pbio.0020422)
Supplement: Table S1 — Also available at http://genes.mit.edu/NielsenEtAl/. (4.3 MB ZIP). [file pbio.0020422.st001.zip › NielsenEtAl/html/237.html]

AN4449.1.NCU03304.1.MG04551.1.FG01236.1


```
 CLUSTAL W (1.82) Multiple Sequence Alignments - Introns Inserted


Sequence 1: NCU03304.1	287 aa
Sequence 2: FG01236.1	191 aa
Sequence 3: MG04551.1	192 aa
Sequence 4: AN4449.1	192 aa
Alignment Length: 288 aa
Number Identitical Residues: 158 aa
Alignment Score (without introns) 6433


MG04551.1 	------------------------~-----------------------------------
NCU03304.1	MYSELSESFHGRRASCKIRRGNYA0WTPPPSSPPPSQPRQLRVALPGPVRLVNWSFPCPF
FG01236.1 	------------------------~-----------------------------------
AN4449.1  	------------------------~-----------------------------------
          	                                                            

MG04551.1 	--~-----------------------------~------MVGKDCVAIACDLRLGMQALT
NCU03304.1	EA1NVPAPSPNITSSSEQATITTCITSQAQDD1GGAAVAMVGKDCVAIACDLRLGLQALT
FG01236.1 	--~-----------------------------~------MVGKDCVAIACDLRLGLQALT
AN4449.1  	--~-----------------------------~------MVGKDCVAIACDLRLGMQALT
          	                                       ****************:****

MG04551.1 	VSNNFPKIFSYSSSVFLGLTGLATDVATVSDLFRYKVNMYRLREERNIAPRTFANLVSSS
NCU03304.1	VSNNFPKIFQYG-DVFLGLTGLATDVSTVSDLFRYKVNMYRLREERNIAPRTFANLVSSS
FG01236.1 	VSNDFPKIFQYG-DVFLGLTGLATDVNTVSDLFRYKVNMYRLREERDIAPRTFANLVSSS
AN4449.1  	VSNNFPKIFNYAPGTYLGLTGLATDVSTVSDLFRLKVNMYRLREERHIAPQTLANLVSST
          	***:*****.*....:********** ******* ***********.***:*:******:

MG04551.1 	LYERRFGPYFVSPVVAGLEPKTGAPFICGFDSIGCIDFAKDFIVSGTASEQLFGMCESLW
NCU03304.1	LYERRFGPYFVSPVVAGLDPKTGEPFICGFDSIGCIDFAKDFIVSGTATEQLFGMCENLW
FG01236.1 	LYERRFGPYFVSPVVAGLDPKTGKPFICGFDSIGCIDFAKDFIVSGTASEQLFGMCEGLW
AN4449.1  	LYERRFGAYFVSPVIAGINNTTGKPFICGFDSIGCIDFAKDFIVSGTASDQLFGTCEGLW
          	*******.******:**:: .** ************************::**** **.**

MG04551.1 	EPDL0GPEDLFETISQALLSAVDRDALSGWGAHVYIIEKDKVTKRLLKGRQD
NCU03304.1	EPNL0GPEDLFETISQALLNAVDRDALSGWGAHVYIIEKDKVTKRLLKGRQD
FG01236.1 	EPDL0EPDALFETISQSLLNAVDRDALSGWGAHVYIIEKDKVTKRLLKGRQD
AN4449.1  	EPDM0SPEDLFETISQALLSAVDRDALSGWGAQVYIIEKDKVTQRLLKGRQD
          	**::  *: *******:**.************:**********:********
```
